# High Prevalence of Hypocalcemia in Non-severe COVID-19 Patients: A Retrospective Case-Control Study

**DOI:** 10.3389/fmed.2020.590805

**Published:** 2021-01-07

**Authors:** Rimesh Pal, Sant Ram, Deepy Zohmangaihi, Indranil Biswas, Vikas Suri, Laxmi N. Yaddanapudi, Pankaj Malhotra, Shiv L. Soni, Goverdhan D. Puri, Ashish Bhalla, Sanjay K. Bhadada

**Affiliations:** ^1^Department of Endocrinology, Post Graduate Institute of Medical Education and Research, Chandigarh, India; ^2^Department of Biochemistry, Post Graduate Institute of Medical Education and Research, Chandigarh, India; ^3^Department of Anesthesiology, Post Graduate Institute of Medical Education and Research, Chandigarh, India; ^4^Department of Internal Medicine, Post Graduate Institute of Medical Education and Research, Chandigarh, India

**Keywords:** COVID-19, calcium, hypocalcemia, hypovitaminosis D, vitamin D

## Abstract

**Purpose:** To compare serum total calcium and phosphate levels in patients with non-severe COVID-19 with age, sex, and serum 25-hydroxyvitamin D level matched healthy adult cohort.

**Methods:** In this retrospective case-control study, medical records of patients (≥18 years) diagnosed as non-severe COVID-19 admitted at and discharged from our tertiary care institution during the period from April 10, 2020 and June 20, 2020 were retrieved. Baseline investigations, notably, serum calcium, phosphate, albumin, magnesium, 25-hydroxyvitamin D, and C-reactive protein (CRP), were performed at admission before any form of calcium or vitamin D supplementation were considered. The biochemical parameters were compared with age, sex, and 25-hydroxyvitamin D matched healthy adult controls (1:1 ratio) derived from the Chandigarh Urban Bone Epidemiological Study (CUBES).

**Results:** After exclusion, 72 patients with non-severe COVID-19 (63 mild and 9 moderate disease) and an equal number of healthy controls were included in the final analysis. Age, sex, serum 25-hydroxyvitamin D, and albumin levels were matched between the 2 groups. Hypovitaminosis D and hypocalcemia were seen in 97 and 67% of the patients, respectively. The patients had lower serum calcium (*P* value <0.001) and phosphate (*P* = 0.007) compared with the controls. There was no statistically significant correlation between serum calcium and CRP.

**Conclusions:** Hypocalcemia is highly prevalent even in COVID-19 patients with non-severe disease probably implying that hypocalcemia is intrinsic to the disease. Prospective studies with larger number of patients are required to prove this hypothesis and unravel the underlying pathophysiological mechanisms.

## Introduction

The novel coronavirus disease (COVID-19) has affected over 50 million people and has inflicted more than 1.2 million casualties ever since its inception in December 2019. Although most patients tend to have mild-moderate disease with a favorable prognosis, the infection may not infrequently lead to severe-to-critical disease and mortality (1). The prognosis tends to be poor, specifically in patients with advancing age and in those with co-morbid conditions (2). Besides, multiple hematological and biochemical parameters have emerged as potential biomarkers to predict severe disease and mortality in COVID-19 (3, 4).

One such biochemical biomarker is hypocalcemia. Hypocalcemia is associated with severe disease, organ failure, increased likelihood of hospitalization, admission to the intensive care unit, need for mechanical ventilation, and death in COVID-19 (5–8). Nevertheless, hypocalcemia is otherwise common in critically ill patients and is associated with disease severity and increased mortality (9–12). Hence, hypocalcemia in COVID-19 patients with severe or critical disease is not unexpected. However, to date, the prevalence of hypocalcemia in non-severe COVID-19 patients has not been explored. In our clinical practice, we had encountered cases of biochemically confirmed hypocalcemia in patients with non-severe COVID-19.

Hence, the present study was undertaken to evaluate serum total calcium and phosphate in patients with non-severe COVID-19 and compare it with age, sex, and serum 25-hydroxyvitamin D level matched healthy adult cohort.

## Materials and Methods

In this clinical retrospective study, we selected patients (aged ≥ 18 years) diagnosed with non-severe COVID-19 (mild and moderate disease) admitted at and discharged from our tertiary care institution during the period from April 10, 2020 and June 20, 2020. Disease severity was based on the clinical management protocol laid down by the Ministry of Health and Family Welfare, Government of India (13). Patients with known chronic kidney disease, parathyroid disorder, calcium/vitamin D supplements over the last 3 months, on anti-osteoporotic medications, glucocorticoids, or anti-epileptic medications were excluded. Baseline serum calcium, phosphate, albumin, magnesium, and 25-hydroxyvitamin D levels measured at hospital admission before any form of calcium or vitamin D supplementations were retrieved from medical records. Besides, baseline C-reactive protein (CRP) was recorded.

As we had included only COVID-19 patients without severe/critical disease, we planned to compare the biochemical profile of the cases with age, sex, and 25-hydroxyvitamin D matched healthy adult cohort (1:1 ratio) derived from the Chandigarh Urban Bone Epidemiological Study (CUBES). The CUBES was undertaken by the authors (RP, SR, SKB) to develop reference data on metabolic bone profile amongst the healthy adult North Indian population (14). Specifically, the study participants were residents of Chandigarh, as were most of the COVID-19 patients included in the index study. Besides, the biochemical investigations of CUBES were also performed at the same institution.

Serum total calcium [reference range (RR) 8.8–10.2 mg/dl], albumin (RR 3.4–4.8 g/dl), inorganic phosphate (RR 2.7–4.5 mg/dl), total alkaline phosphatase (RR 40–129 IU/l), creatinine (RR 0.6–1.2 mg/dl), magnesium (RR 1.7–2.2 mg/dl) and CRP (RR <5 mg/ l) were measured by auto-analyzer (Roche diagnostics, Cobas C702, Germany). Calcium values were adjusted for the respective serum albumin levels. Serum 25-hydroxyvitamin D (RR 11.2–42.8 ng/ml) was measured by electrochemiluminescence immunoassay (Roche diagnostics, Elecsys 2010 system, Germany).

The study was approved by the Institute Ethics Committee, Post Graduate Institute of Medical Education and Research, India.

### Statistical Analysis

Statistical analysis was performed using Statistical Package for Social Sciences (SPSS) 23.0 software (SPSS Inc., Chicago, IL, USA). Kolmogorov-Smirnov test was used to check the normality of data. The normally distributed data were expressed as mean ± standard deviation (SD), while non-parametric data were expressed in median (interquartile range, IQR). Vitamin D deficiency and severe vitamin D deficiency were defined as serum 25-hydroxyvitamin D <20 and <10 ng/ml, respectively (15). Hypocalcemia was defined as serum total albumin-adjusted calcium <8.8 mg/dl (16). The comparison of presenting symptoms between patients with mild and moderate disease was made using the Chi-Square test or Fisher's Exact test. Likewise, the comparison in biochemical parameters between case and control groups were made using Independent Samples *t*-Test or Mann-Whitney *U* tests based on normality of data. Correlations between biochemical parameters were established using Spearman rank-order correlation. A *P* < 0.05 was considered significant.

## Results

The medical records of 82 patients admitted and discharged with the diagnosis of non-severe COVID-19 in the stipulated time were retrieved. Of these 82 patients, 10 patients were excluded (3 due to age <18 years, 7 due to incomplete medical records). Finally, 72 patients with non-severe COVID-19 with complete medical records were included in the final analysis. A similar number of healthy adult men and women were recruited from CUBES for comparison. Only nine patients (12.5%) had moderate disease, while the rest (87.5%) had mild disease. The presenting symptoms of patients with mild and moderate disease have been represented in Table 1. In short, patients with moderate disease were more likely to have fever, cough, and shortness of breath at presentation than patients with mild disease. Eleven patients (15%) had a history of type 2 diabetes mellitus.

**Table 1:** Showing presenting symptoms of non-severe COVID-19 patients with mild and moderate disease.

**Presenting symptom**	**Mild disease (*n* = 63)**	**Moderate disease (*n* = 9)**	***P***
Fever	41 (65%)	9 (100%)	0.033^*^
Sore throat	31 (49%)	2 (22%)	0.166^#^
Cough	27 (43%)	9 (100%)	0.001^*^
Shortness of breath	0 (0%)	9 (100%)	**<0.001**^#^
Generalized weakness	37 (59%)	8 (89%)	0.080^*^
Anosmia	9 (14%)	1 (11%)	1.000^#^
Ageusia	8 (13%)	1 (11%)	1.000^#^
Diarrhea	11 (18%)	3 (33%)	0.363^#^

* *By Chi-Square test*.

# *By Fisher's Exact test*.

**Figure 1:**
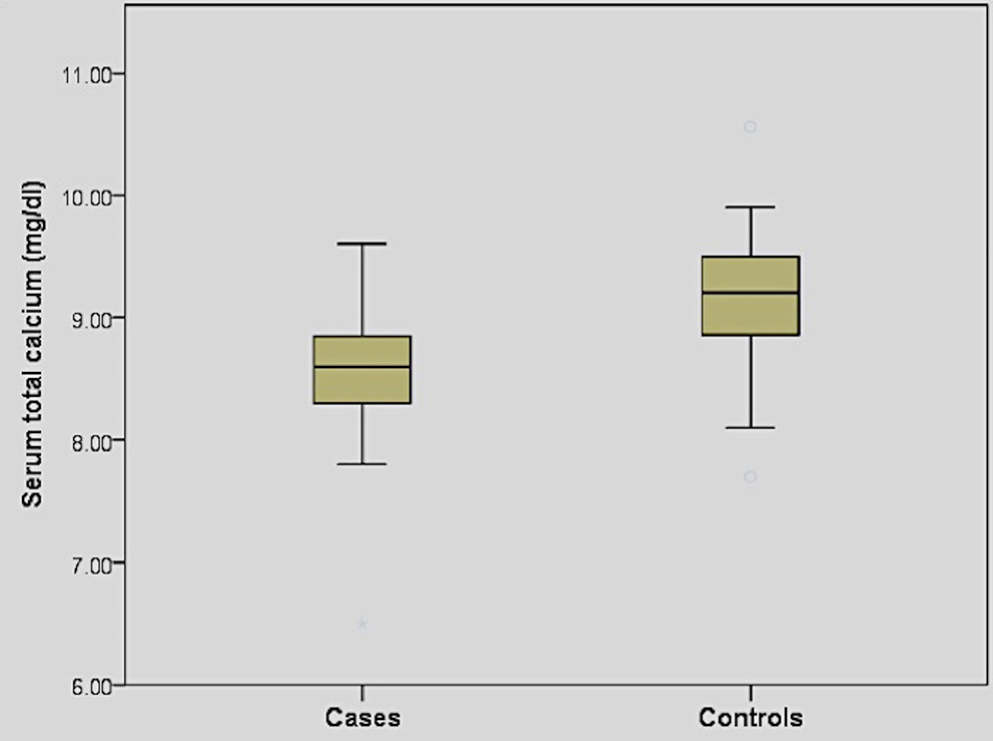
Box and whisker plot showing comparison of serum total calcium in non-severe COVID-19 patients (cases) and healthy controls.

The baseline demography and biochemical characteristics of patients and controls have been summarized in Table 2. The age, sex, serum 25-hydroxyvitamin D, and albumin levels were matched between the case and control groups. Seventy patients (97%) in the COVID-19 group had vitamin D deficiency, while 43 patients (60%) were severely vitamin D deficient. Similarly, hypocalcemia was highly prevalent in the COVID-19 group seen in 48 patients (67%). There was no gender difference in serum calcium in the COVID-19 group (*P* = 0.569). Serum calcium levels did not differ between patients with mild and moderate disease (*P* = 0.099). When compared with the control group, serum total calcium was lower in the COVID-19 group (*P* < 0.001) (Figure 1). Similarly, serum phosphate was also lower in the COVID-19 group than the control group (*P* = 0.007) (Table 2).

**Table 2:** Showing comparison of demographic and baseline biochemical parameters between non-severe COVID-19 patients (cases) and healthy controls.

**Parameters**	**Non-severe COVID-19 patients (*n* = 72)**	**Matched healthy controls from CUBES (*n* = 72)**	***P***
**Age (years)**			
Mean ± SD Median (IQR)	37.5 ± 13.7 36.0 (27.0–48.25)	36.9 ± 13.7 35.0 (25.2–45.2)	–
Male:Female ratio	1.7:1.8	1.7:1.8	–
**Serum 25(OH)D (ng/ml)**			
Mean ± SD Median (IQR)	9.8 ± 4.5 9.1 (6.8–12.3)	9.6 ± 4.4 8.8 (6.7–11.4)	–
**Serum creatinine (mg/dl)**			
Mean ± SD Median (IQR)	0.72 ± 0.20 0.70 (0.60–0.85)	0.73 ± 0.17 0.71 (0.58–0.85)	0.619^*^
**Serum albumin (g/dl)**			
Mean ± SD Median (IQR)	4.3 ± 0.6 4.4 (4.1–4.7)	4.3 ± 0.3 4.2 (4.1–4.5)	0.999^#^
**Serum albumin-adjusted total calcium (mg/dl)**			
Mean ± SD Median (IQR)	8.6 ± 0.4 8.6 (8.3–8.9)	9.1 ± 0.5 9.2 (8.8–9.5)	**<0.001**^#^
**Serum non-adjusted total calcium (mg/dl)**			
Mean ± SD Median (IQR)	8.8 ± 0.3 8.8 (8.5–9.2)	9.3 ± 0.4 9.3 (9.0–9.7)	**<0.001**^#^
**Serum phosphate (mg/dl)**			
Mean ± SD Median (IQR)	3.3 ± 0.7 3.3 (2.9–3.7)	3.6 ± 0.5 3.6 (3.2–3.9)	**0.007**^#^
**Serum total ALP (IU/l)**			
Mean ± SD Median (IQR)	103.2 ± 38.1 93.5 (78.2–123.7)	100.2 ± 26.3 96.0 (83.2–112.0)	0.771^*^
**Serum magnesium (mg/dl)^∧^**			
Mean ± SD Median (IQR)	2.1 ± 0.2 2.1 (1.9–2.2)	NA NA	–
**Serum CRP (mg/l)**			
Mean ± SD Median (IQR)	21.6 ± 62.6 3.0 (1.1–8.8)	NA NA	–

* *By Mann-Whitney U test*.

# *By Independent Samples t-Test*.

∧ *Baseline data on serum magnesium was available in only 65 patients*.

*COVID-19, Novel coronavirus disease; CUBES, Chandigarh Urban Bone Epidemiological Study; SD, Standard deviation; IQR, Interquartile range; 25(OH)D, 25-hydroxyvitamin D; ALP, Alkaline phosphatase; CRP, C-reactive protein; NA, Not available*.

There was no statistically significant correlation between serum total calcium and CRP (r_s_ = 0.063, *P* = 0.599), 25-hydroxyvitamin D (r_s_= −0.119, *P* = 0.320), magnesium (r_s_ = −0.033, *P* = 0.792) or total ALP (r_s_ = 0.158, *P* = 0.184). Similarly, CRP did not correlate with serum phosphate (r_s_ = −0.020, *P* = 0.869), 25-hydroxyvitamin D (r_s_ = 0.062, *P* = 0.605), or magnesium (r_s_ = 0.059, *P* = 0.641).

## Discussion

In this retrospective case-control study, we found that COVID-19 patients with non-severe disease had lower serum total calcium and phosphate levels than age, sex, and 25-hydroxyvitamin D matched controls. Hypocalcemia and hypovitaminosis D were prevalent in 67 and 97% of the COVID-19 patients, respectively.

Hypocalcemia is common in critically ill patients. Vitamin D deficiency and/or resistance (17, 18), acquired “relative” hypoparathyroidism, and 1α-hydroxylase deficiency are the proposed mechanisms leading to hypocalcemia in critically ill patients (17). Cytokines, notably interleukin (IL)-1β and IL-6, lead to an upregulation of calcium-sensing receptor (CaSR) on parathyroid glands, increasing the sensitivity of the parathyroid cells to circulating calcium, resulting in a state of acquired relative hypoparathyroidism (19, 20). Other causes of hypocalcemia in the critical care setting include hypoalbuminemia, fluid overload (dilutional hypocalcemia), alkalosis, acute renal insufficiency, chelation after pheresis, or transfusion of citrated blood and drug-induced (9).

Hypocalcemia is also seen in several viral infections, such as severe acute respiratory syndrome (SARS), avian influenza, and Ebola virus disease. Hypocalcemia has also been documented in patients with COVID-19 (5–8, 21). However, most of the available studies have included only/mostly patients with severe/critical disease (5–7). On the other hand, in the rest of the studies, data on disease severity has not been presented (8, 21). As hypocalcemia is common in critically ill patients, it is not surprising that severe/critically ill patients with COVID-19 had a high prevalence of hypocalcemia. Cappellini et al. compared calcium profile in 420 patients with COVID-19 and 165 patients without COVID-19; they found that COVID-19 patients had significantly low serum total calcium and whole blood actual ionized calcium compared with non-COVID-19 patients. However, whether the two groups were matched in terms of disease severity was not presented (21). Moreover, most of the aforementioned studies did not assess serum 25-hydroxyvitamin D levels, which could have been an underlying confounding factor, partly contributing to hypocalcemia (5, 6, 8, 21). Sun et al. had measured serum 25-hydroxyvitamin D in only 26 (out of 241) patients with COVID-19 and found a positive correlation between serum calcium and 25-hydroxyvitamin D levels. Besides, all 26 patients were found to have vitamin D deficiency (7).

Keeping all these confounding factors in mind, we planned to estimate serum total corrected calcium levels in non-severe (mild and moderate) COVID-19 patients, thereby ruling out the possibility of any critical illness-induced alteration in calcium dynamics. Also, we assessed the serum 25-hydroxyvitamin D levels in all the patients at initial admission and compared the biochemical profile of the patients with age, sex, and 25-hydroxyvitamin D level matched healthy adult cohort. Still, we found that non-severe COVID-19 patients had significantly lower serum calcium levels than healthy counterparts. The data supports the fact that hypocalcemia is *probably intrinsic* to COVID-19. However, as shown in prior studies, the degree of hypocalcemia depends on the severity of the disease (5–8).

Hypocalcemia in COVID-19 could be a direct effect of SARS-CoV-2 or could result from an imbalance in parathyroid hormone (PTH) and/or 25-hydroxyvitamin D. In general, calcium is required for virus structure formation, entry, replication, and virion release. *In vitro* and animal studies with SARS-CoV (responsible for the SARS outbreak in 2003) had shown that the viral E gene encodes a small transmembrane protein that functions as a calcium ion channel. It was also demonstrated that alteration in calcium homeostasis within the host cell could lead to the activation of inflammatory pathways resulting in lung cell damage (22). Considering the remarkable similarity between SARS-CoV-2 and SARS-CoV genomes, the same mechanism might be operational in COVID-19 as well (21). Besides, elevated levels of unbound and unsaturated fatty acids have been reported in COVID-19 patients. Unsaturated fatty acids can bind calcium, causing hypocalcemia (23, 24). An alternate explanation could be a reduction in renal 1α-hydroxylase activity in patients with COVID-19. Angiotensin-converting enzyme 2 (ACE2), the receptor for the SARS-CoV-2, is expressed in the proximal renal tubule, the predominant site of expression of renal 1α-hydroxylase. The impaired renal 1α-hydroxylase activity would reduce calcitriol production that could explain both hypocalcemia and hypophosphatemia.

The overall occurrence of hypovitaminosis D (97%) in the COVID-19 patients deserves a special mention. The prevalence of hypovitaminosis D and severe hypovitaminosis D in the CUBES was 65.4 and 37.5%, respectively (14). The high prevalence in the cases could be explained based on the nationwide lockdown that had possibly limited the duration of sunlight exposure and thereby *in-vivo* generation of vitamin D. Alternatively, it can be hypothesized that hypovitaminosis D, *per se*, is a risk for incident COVID-19 infection (25). In a retrospective case-control study conducted in Northern Spain, the prevalence of hypovitaminosis D in hospitalized patients with COVID-19 was 82.2% while that in population-based controls was 47.2% (26).

We do respect the limitations of the study. First, the sample size was relatively small. Second, the number of COVID-19 patients with moderate disease was very few; hence, the lack of difference in serum calcium levels between patients with mild and moderate disease might not be statistically valid. Indeed, the study was underpowered to delineate any significant difference in serum calcium levels between patients with mild and moderate disease (power being 44.14%). Third, we considered serum total corrected calcium levels instead of ionized calcium values. Serum total calcium might be affected by blood pH; however, as all the patients were non-severe (the majority being mild in severity), we believe that alteration in blood pH would not have been an issue in the cases. Fourth, we did not measure PTH or calcitriol levels in our patients to lend support to our aforementioned hypotheses. Last, as all the patients had non-severe disease and were discharged with an uneventful in-hospital course, we did not correlate the patients' final outcome with the biochemical parameters.

## Conclusions

Patients with non-severe COVID-19 tend to have low serum total calcium levels at initial presentation, implying that hypocalcemia is probably intrinsic to the disease *per se*. However, large-scale prospective studies are required to prove this hypothesis and unravel the underlying pathophysiological mechanisms.

## Data Availability Statement

The raw data supporting the conclusions of this article will be made available by the authors, without undue reservation.

## Ethics Statement

The studies involving human participants were reviewed and approved by Post Graduate Institute of Medical Education and Research, Chandigarh, India. Written informed consent for participation was not required for this study in accordance with the national legislation and the institutional requirements.

## Author Contributions

RP is the primary author. SR and SB had conceptualized and had edited the manuscript. DZ, IB, VS, LY, SS, PM, and AB had helped in data collection. GP had edited the manuscript. All the authors approved the final version of the manuscript.

## Conflict of Interest

The authors declare that the research was conducted in the absence of any commercial or financial relationships that could be construed as a potential conflict of interest.
